# GENOVIS: a Python package for the visualization of population genetic analyses

**DOI:** 10.1186/s12864-026-12598-x

**Published:** 2026-02-10

**Authors:** Siavash Salek Ardestani, Elmira Mohandesan

**Affiliations:** 1 Department of Interdisciplinary Life Sciences, Konrad Lorenz Institute of Ethology, University of Veterinary Medicine, Savoyenstrasse 1, 1160 Vienna, Austria; 2 Department of Evolutionary Anthropology, University of Vienna, Djerassiplatz 1, 1030 Vienna, Austria; 3 Human Evolution and Archaeological Sciences (HEAS), University of Vienna, Djerassiplatz 1, 1030 Vienna, Austria

**Keywords:** Data visualization, Population genomics, Publication-ready graphics, Bioinformatics software, SNP density, Runs of homozygosity, Principal component analysis, Admixture, Genomic relationship heatmap, Manhattan plot

## Abstract

**Background:**

Despite its importance, generating clear and customisable figures remains challenging for researchers without a bioinformatic background. This is due to the fact that most population genetics tools target specific analyses and rely on language-specific scripting (e.g., R or Python), producing large amounts of non-interactive outputs. Moreover, many visualization tools are challenging to integrate into existing analytical pipelines or cross-platform environments, adding time-consuming and technically demanding steps. Therefore, there is a growing demand for powerful, user-friendly, and flexible visualization tools that enable researchers with varying levels of bioinformatic expertise to investigate and communicate a wide range of population genetic questions, using both simulated and empirical datasets.

**Results:**

To address this need, we developed GENOVIS, a Python package available both as a command-line and graphical interfaces (CLI and GUI) that streamlines and simplifies the generation of six key population genomics visualizations: single-nucleotide polymorphism (SNP) density heatmaps (*mapden*), runs of homozygosity (ROH) plots (*rohpainter*), genomic relationship matrix heatmaps (*relmap*), 3D principal component analysis (PCA) (*pca3d*), admixture barplots (*admix*), and Manhattan plots (*manplot*). As such, GENOVIS provides a unified and user-friendly interface to produce publication-ready figures with minimal coding and high flexibility.

**Conclusion:**

With the development of the visualization software GENOVIS, we provide a streamlined solution for generating high-quality, publication-ready graphics with customizable features through both CLI and GUI interfaces. Built on a flexible Python framework, GENOVIS enables efficient generation of runs of homozygosity plots, 3D PCA, admixture barplots, SNP density heatmaps, Manhattan plots, and genomic relationship heatmaps, making advanced population genomics analyses more accessible and reproducible.

**Supplementary Information:**

The online version contains supplementary material available at 10.1186/s12864-026-12598-x.

## Background

With the advent of high-throughput DNA sequencing technologies, the field of population genetics benefits from a large amount of genomic data that enables more comprehensive analyses [1, 2]. These studies heavily rely on effective visualization, which is essential for interpreting various population genetics key concepts. Widely used and informative analyses such as admixture analysis, PCA, Genome-wide association study (GWAS), and measures of genetic diversity can be complex and challenging to interpret without clear visualization [3–5].

The critical role of visualization is evident in numerous studies across a range of species, including humans [6, 7], sheep [8, 9], cattle [10, 11], rice [12, 13], and wheat [14, 15]. To support these insights, researchers routinely apply a variety of visualization tools, each tailored to the study’s objectives and offering distinct perspectives on various dynamics in species’ evolutionary history. Central to many of these analyses are SNP datasets, which are cost-effective and informative for a wide range of population genetic analyses, such as GWAS, ROH, admixture, and PCA [16–19].

Building on the importance of SNP datasets, population genomics studies often analyse genome-wide SNP density to investigate genome structure and the influence of various evolutionary forces. For example, detecting genomic regions with unexpectedly high or low SNP densities may indicate various evolutionary footprints [20, 21]. Analysing SNP densities within defined bin sizes improves downstream analyses, particularly the detection of ROHs with tools like PLINK [22, 23]. ROHs, continuous homozygous regions inherited from both parents [24], provide valuable insights into population history and can identify potential signatures of selection when visualized across the genome [25]. To the best of our knowledge, there are currently two R packages available for visualizing ROH regions [26, 27].

Similar to the ROH, genomic relationship matrices are informative measures in population genomics studies, assessing genetic similarity between individuals and uncovering population structure [28]. Visualizing these matrices as heatmaps allows for quick identification of clustering patterns, genetic relatedness, and outliers. A genomic relationship matrix can also be used (as a covariance matrix) in PCA, reducing high-dimensional genomic data into key components, which highlight genetic clusters and differentiation [29]. Typically, PCA results are visualized in two-dimensional (2D) scatter plots. However, adding a third dimension can reveal an additional layer of population structure that might not be detected in a 2D PCA plot [30]. Several packages in R (e.g., ggplot2) and Python (e.g., Matplotlib) support 3D PCA plotting; however, implementing these tools often requires custom-made scripts, which can be challenging and not user‑friendly for all users. While PCA offers a geometric view of population differentiation, admixture analysis [31] offers a complementary perspective by estimating genetic ancestry proportions. Tools such as ADMIXTURE [31], NGSremix [32], FastNGSadmix [33], and frappe [34] are widely used for admixture analysis; however, they lack built-in graphical output. In many GWAS [35–39] and selection signature studies [8, 40–42], PCA and admixture analysis have been routinely performed to investigate the population structure. Manhattan plots are standard for the visualization of GWAS and selection signature results. Several previously developed R packages are available for creating Manhattan plots, including CMplot [43] and qqman [5]. However, similar to other plots mentioned above, generating Manhattan plots often requires customized scripting and/or multiple tools, which limit accessibility for some users. Visualization plays a vital role in population genetics analyses, as supported by numerous publications. However, the lack of integration among existing tools often results in inefficient workflows and limits comprehensive interpretation. This highlights the need for integrative, user-friendly platforms that streamline visualization and improve data analysis and interpretation.

## Implementation

**Fig. 1:**
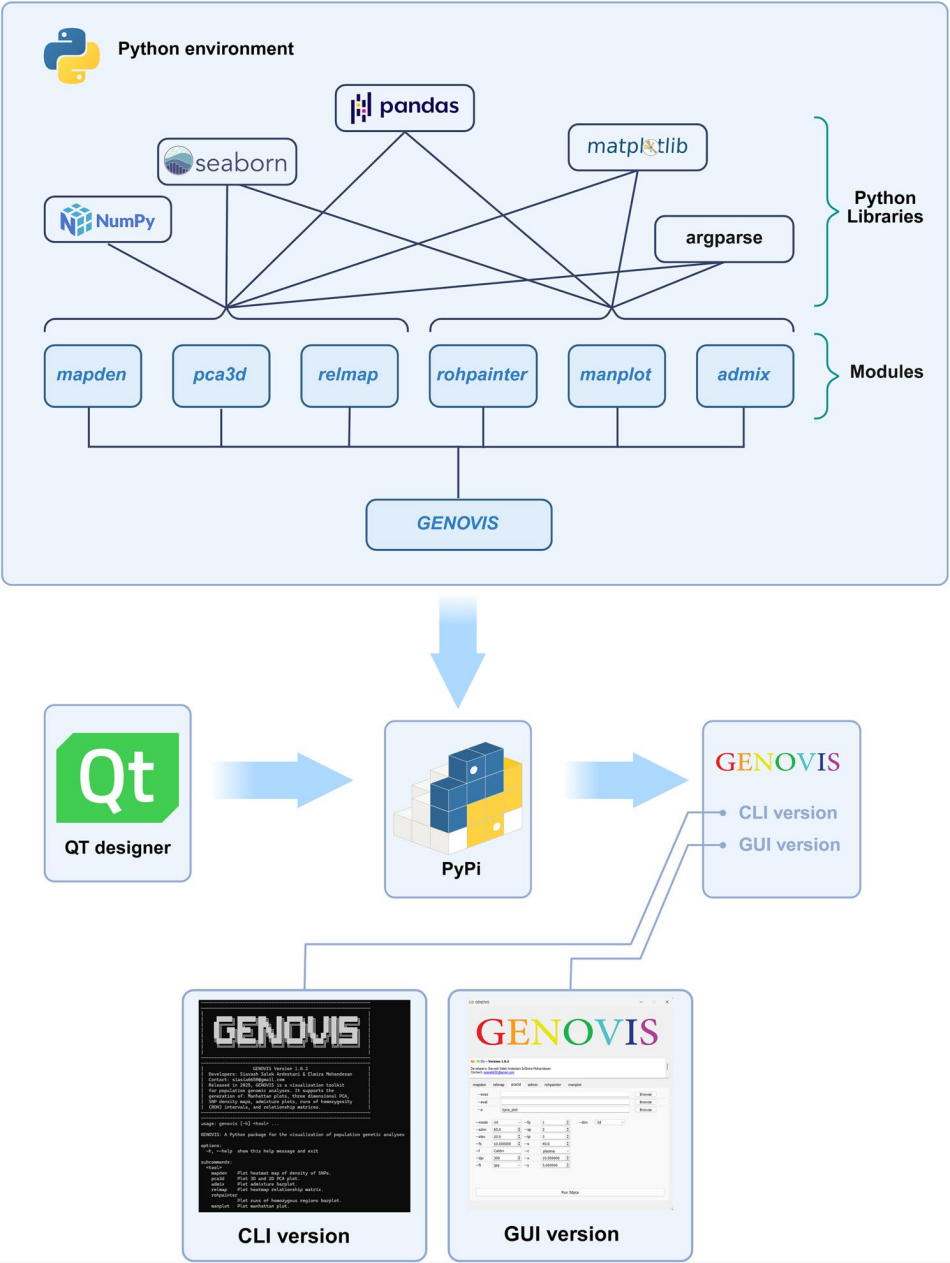
GENOVIS structure for both command-line interface (CLI) and graphical user interface (GUI), and module organisation, along with Python libraries used for implementation: matplotlib, argparse, numpy, pandas, and seaborn

GENOVIS is a package (available both as CLI and GUI) for visualizing results of different population genomic analyses, including: SNP densities (*mapden*), ROH (*rohpainter*), relationship matrix (*relmap*), PCA (*pca3d*), admixture (*admix*), and Manhattan plots (*manplot*). To develop GENOVIS in a Python 3.11.9 environment, we employed Python libraries (Fig. 1) such as matplotlib 3.10.3 [44], argparse 1.1, numpy 2.2.5 [45], pandas 2.2.3 [46], and seaborn 0.13.2 [47]. Additionally, the GUI version of GENOVIS was designed using Qt Designer 5.11.1 (https://doc.qt.io/archives/qt-5.15/qtdesigner-manual.html), providing an interactive shell for streamlined user interaction. The designed shell was saved as a *.ui file, and subsequently converted to a Python script using the pyuic5 5.15.10 software (https://www.riverbankcomputing.com/software/pyqt/intro). Finally, GENOVIS was implemented as a fully modular Python package and distributed via the Python Package Index (PyPI) (https://pypi.org/project/genovis). This packaging approach provides cross-platform compatibility, automatic dependency management, reproducible versioned releases, and long-term sustainability of the software (https://pypi.org).

In this study, we utilized a publicly available human [1], horse [40], cattle [10, 48–51], and goat [52] datasets (Table 1) to test GENOVIS modules (see Supplementary information 1).

**Table 1:** Descriptions of publicly available human, horse, cattle, and goat datasets utilized for visualization using GENOVIS

Plot	Species	Number of populations	Population size	Reference
SNP density heatmap	Horse (*Equus caballus*)	-	-	https://webserver.ibba.cnr.it/SNPchimp
Runs of Homozygosity	Cattle (*Bos taurus and Bos indicus*)	7	184	Gao et al. (2017) [48], Matukumalli et al. (2009) [49], Iso-Touru et al. (2016) [50], and Gautier et al. (2009) [51]
Relationship matrix	Horse (*Equus caballus*)	4	169	Mousavi et al. (2023) [40]
PCA	Goat (*Capra hircus*)	2	120	Chen et al. (2022) [52]
Admixture	Human (*Homo sapiens*)	26	2,504	Auton et al. (2015) [1]
Manhattan	Cattle (hybrid *Bos taurus/indicus*)	1	1,218	Forutan et al. (2024) [10]

## Results and discussion

### SNP density heatmap module (*mapden*)

**Fig. 2:**
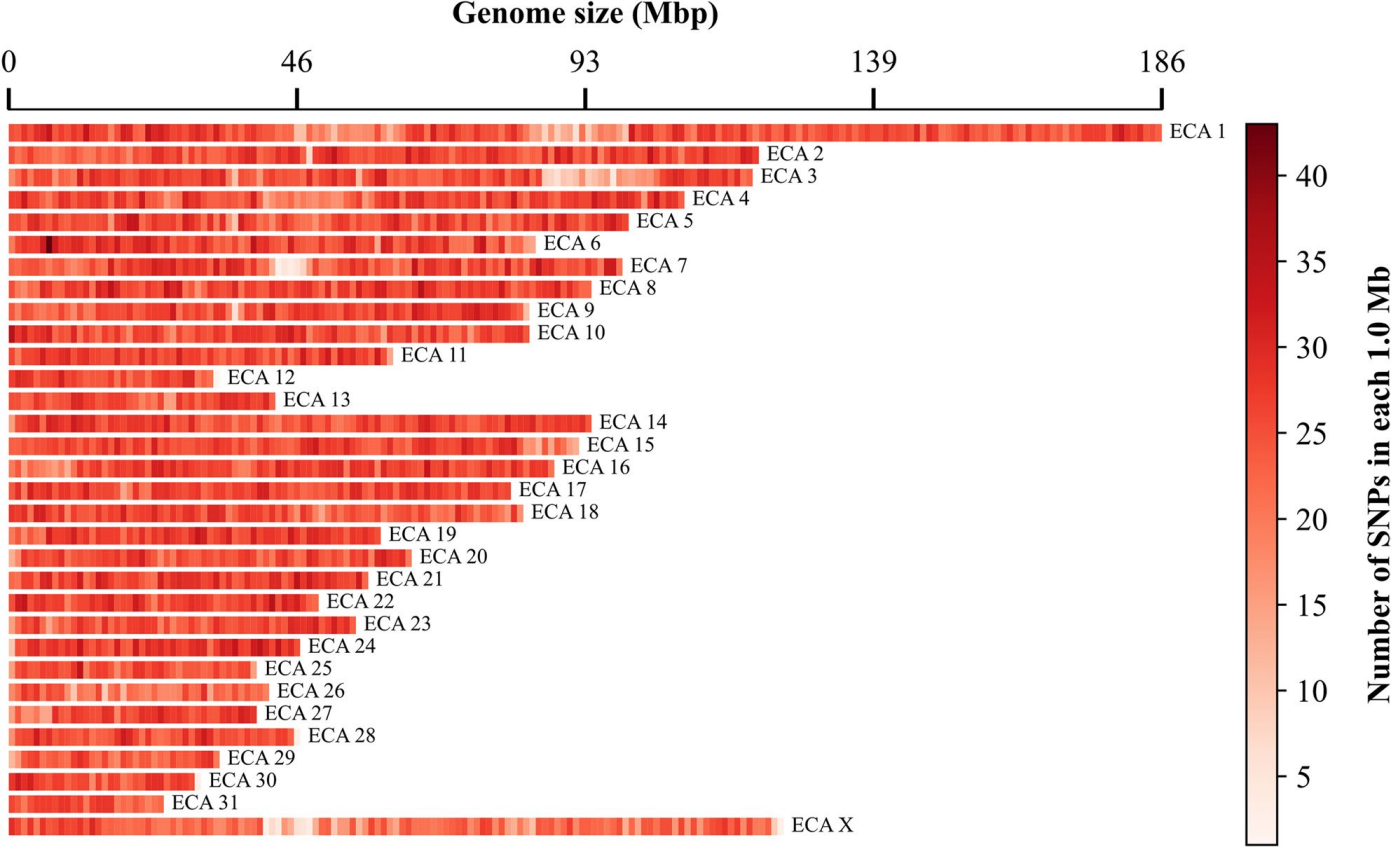
Horse (*Equus caballus*) genome-wide SNP density heatmap based on 65,157 SNPs from the GeneSeek Equine SNP 65 BeadChip (assembly: EquCab 2.0). Chromosomes (rows) are divided into non-overlapping 1 Mb bins, with color intensity indicating the number of SNPs in each bin. Input files include a PLINK-formatted map file and a genome index file (containing chromosome identifiers and their lengths)

SNP density typically refers to the number of SNPs genotyped within a specific genomic interval. The importance of investigating SNP density has been described in previous studies [53, 54]. A heatmap visualization of SNP density summarizes the distribution of genetic markers across the genome and highlights regions with locally increased or reduced SNP density. Such patterns can reflect underlying genomic features, including recombination landscapes. Accordingly, this visualization supports the interpretation of genome-wide variation in population-genomic analyses. To the best of our knowledge, currently there is only one R package (CMplot) [43] for visualizing SNP density (https://github.com/YinLiLin/CMplot). In the GENOVIS package, the *mapden* module is designed to visualize genome‑wide SNP density, using heatmaps across chromosomes (Fig. 2). This module calculates SNP densities by dividing each chromosome into non-overlapping, fixed-size bins (customizable via the “--b” parameter), and calculating the number of SNPs within each bin. The resulting SNP densities are then displayed as individual heatmap tracks per chromosome, where color intensity represents SNP density within each interval.

To generate a genome-wide SNP density map, the input dataframe must conform to the PLINK format (*.map). Moreover, the *mapden* module requires a genome index file (as a second input file), containing the physical length of each chromosome. Unlike CMplot [43], which defines the chromosome size based on the physical position of the last genotyped SNP, *mapden* uses the actual chromosome lengths from the index file mentioned above. This is an important feature in visualization, as it allows for a more accurate representation of a genomic scale.

### Runs of homozygosity visualization module (*rohpainter*)

**Fig. 3:**
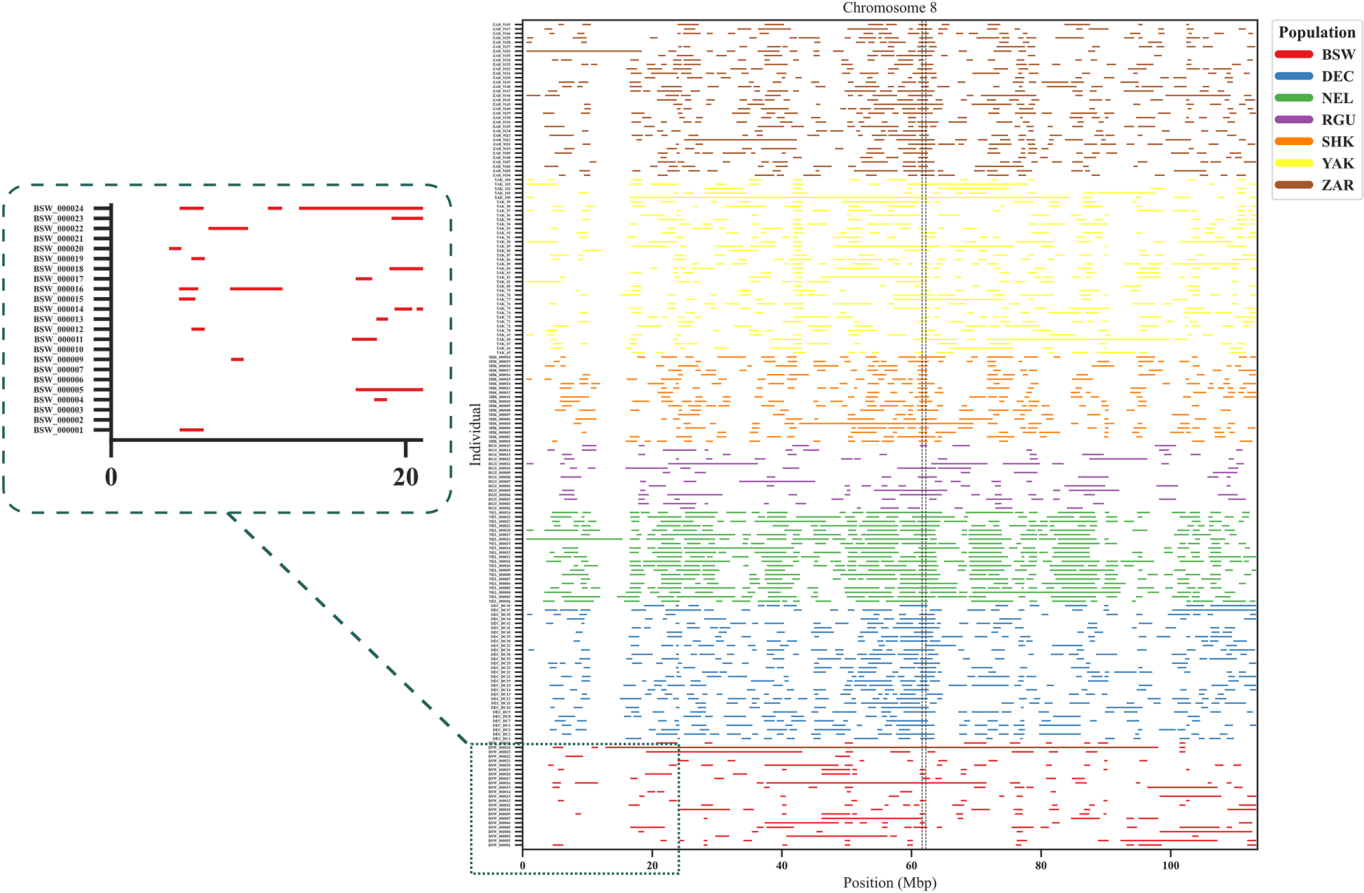
Visualization of Runs of Homozygosity (ROH) of seven cattle (*Bos taurus* and *Bos indicus*) populations (*n* = 184) based on a total number of 27,580 SNPs remained after removing SNPs with minor allele frequency < 0.01, calling rate < 0.90, and Hardy-Weinberg equilibrium P-value < 10^− 6^, using *rohpainter* module. ROH distribution across chromosome 8 in the Brown Swiss (BSW, *n* = 24), Dengchuan (DEC, *n* = 31), Nelore (NEL, *n* = 21, three individuals were removed due to their rate of missing genotype > 0.1 from a total number of 24 Nelore cattle), Red Angus (RGU, *n* = 15), Sheko (SHK, *n* = 20), Yakutian (YAK, *n* = 40), and Arabic Zebu (ZAR, *n* = 35) [48–51]. Each horizontal bar represents an individual’s homozygous segment; black vertical lines indicate common ROH regions shared by ≥ 70% of individuals

The *rohpainter* module enables robust and detailed visualization of ROH across the genome (Fig. 3). Such visualizations provide insight into population-level demographic processes, including inbreeding history and recent population bottlenecks. The genomic distribution and sharing of ROH segments across individuals can highlight autozygous regions shaped by demographic history or selection. In addition to ROH, this module can also be applied to other interval-based genomic data, such as copy number variants (CNVs). Inputs to this module include: (1) genome index (“--i”: chromosomes and sizes) and (2) main data frame (“--d”; population label, Individual ID, chromosome number, start position, and end position). A notable feature of *rohpainter* is the ability to pinpoint and detect common genomic intervals among individuals by applying “--t” (cutoff threshold for minimum frequency of common regions) option (Fig. 3), which has a similar function to the “threshold” option of “tableRuns” in detectRUNS package [26]. By applying this option, a *shared_intervals.txt file will be saved as a dataframe including common genomic intervals among individuals. Setting a minimum frequency threshold for common ROH regions enables the identification of genomic regions potentially under selection pressures in a population [55, 56]. The width and color of threshold lines are customizable by using “--tw” and “--tc” options in *rohpainter*, respectively. Additionally, users can hide or show individual labels by using the “--sl (false/true)” option.

### Genomic relationship visualization module (*relmap*)

**Fig. 4:**
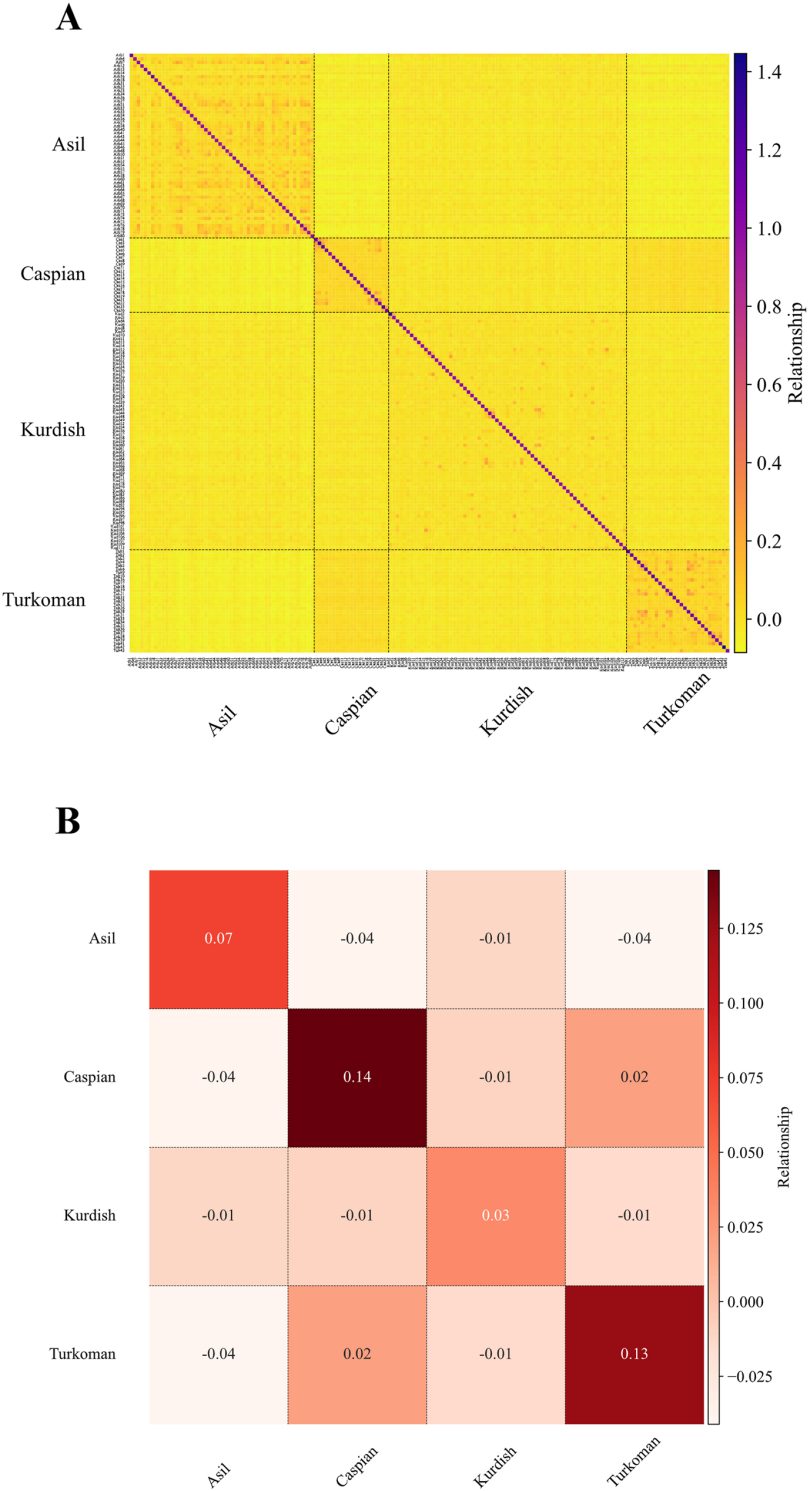
Relationship matrix plot generated using the *relmap* module from GENOVIS package. **A** Heatmap of genomic relationships among individuals (*n* = 169) from four Persian horse (*Equus caballus*) populations, including: Asil (*n* = 52), Caspian (*n* = 21), Kurdish (*n* = 67), and Turkoman (*n* = 29). The relationship matrix was constructed based on quality-controlled (40,120 SNPs) published SNP-array data (GGP Equine 70 K SNP Bead Chip array) by Mousavi et al. [40]. **B** Heatmap of the average genomic relationships within and between populations. Averages of relationships were calculated by applying the “--av true” option, and then annotated with “--a true”

The *relmap* enables the visualization of genomic relationship heatmaps using two input formats, which correspond to outputs generated by various software [22, 57–60]. Genomic relationship matrices summarize genome-wide genetic similarity among individuals and can reveal population structure through clustering patterns. Visual inspection of these heatmaps facilitates detection of closely related individuals or populations and outliers, which is critical for downstream population-genomic analyses. The *relmap* module supports both matrix (“--rf mat”) and columnar (“--rf col”) formats, ensuring compatibility with widely used programs such as PLINK 2.0 [57], PLINK 1.9 [22], and AGHmatrix 2.1.4 package [59]. Users can customize visual elements by enabling or disabling axis labels (“--sl”), adjusting font sizes (--xyfs), specifying color maps (--c), and modifying line widths to suit presentation requirements. An additional key feature of this module is its ability to calculate average pairwise relationships between and within populations using the “--av true” option. Average pairwise relationships can also be used to plot a heatmap relationship at the population level, enabling a simplified and more interpretable visualization of relatedness across population groups. This option is particularly beneficial when working with large datasets where individual-level matrices may be too dense to interpret directly. Additional options include masking diagonal elements (“--mask” true/false) and annotating relationship values within heatmap blocks (“--a” ) (Fig. 4).

### 3D population clustering visualization module (*pca3d*)

**Fig. 5:**
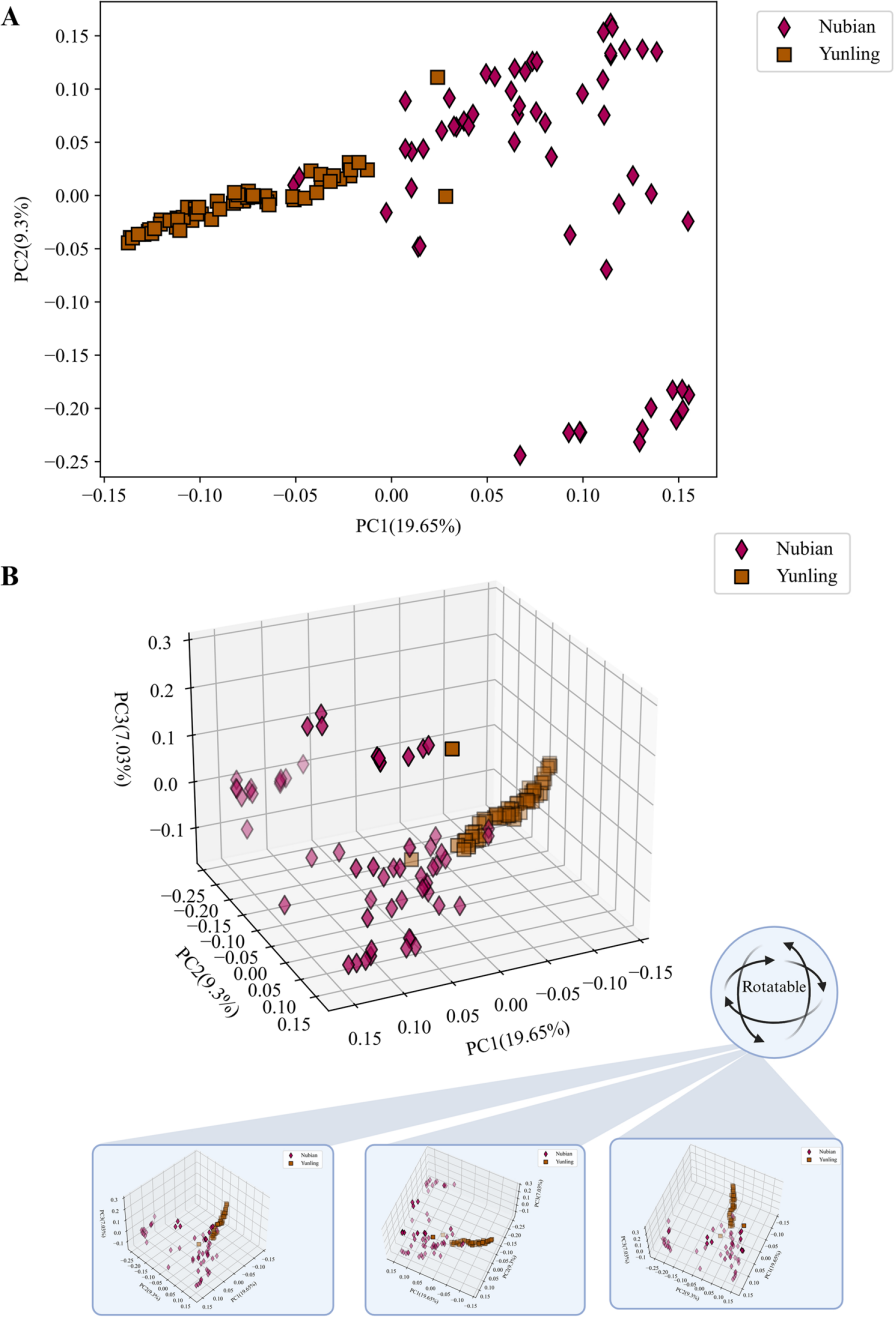
**A** Two- and **B** three-dimensional principal component analysis (2D and 3D PCA) visualization for a total number of 120 published genomic PCA results from two different goat (*Capra hircus*) populations (Nubian and Yunling) [52] using *pca3d* module from the GENOVIS package. The upper panel shows a 3D scatter plot of individuals based on the first three principal components (PC1, PC2, and PC3). The lower panel displays snapshots of the same 3D PCA plot from three different angles

PCA provides a low-dimensional representation of genome-wide variation that is widely used to explore population structure and genetic differentiation. Extending PCA to three dimensions can reveal additional structure not evident in 2D projections and can help identify subtle clusters or outliers [30]. Such visualization supports biological interpretation by clarifying relationships among individuals or groups across multiple principal components [61]. The *pca3d* module visualizes PCA results in three dimensions, and supports two output modes: “--mode interactive and “--mode solid”. The interactive mode allows users to freely rotate the plot by adjusting azimuth and elevation angles in real-time (Fig. 5). In solid mode, these angles can be manually set using “--elev” and “--azim” options. The module requires two input files: eigenvectors (“--evec *.evec”) and eigenvalues (“--eval *.eval”), formatted according to PLINK 1.90 outputs (“--pca/--mds” ) [22]. The *pca3d* module can not only visualize PCA results in three dimensions, but also in two dimensions (Fig. 5) by “--dim 2d” and defining first and second PCs (“--fp” and “--sp”). While PCA typically emphasizes the first few PCs; additional PCs may reveal subtle population structure [62] or outliers [63]. In *pca3d*, the “--fp”, “--sp”, and “--tp” (third PC) options allow users to change PCs for plotting PCA results. The “--s” option is defined to adjust the size of scatter points.

### Ancestry proportion visualization module (*admix*)

**Fig. 6:**
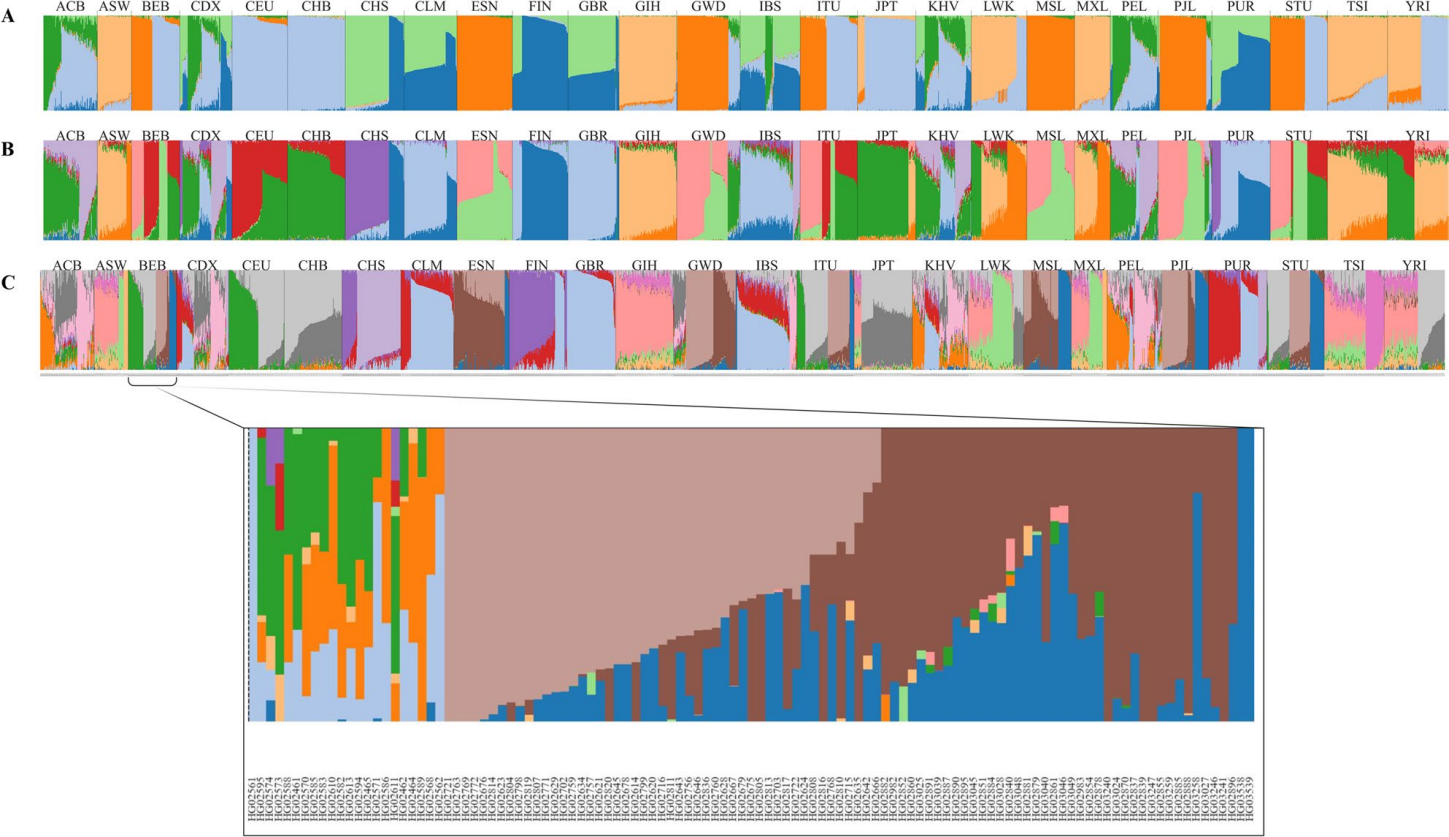
Admixture plot generated by the *admix* module from the GENOVIS package, based on 2,504 published admixture results (1000genome project: https://ftp.1000genomes.ebi.ac.uk/vol1/ftp/release/20130502/supporting/admixture_files.) from 26 human (*Homo sapiens*) populations (*n* = 2,504) including: African Caribbean (ACB, *n* = 96), African Ancestry Southwest (ASW, *n* = 61), Bengali (BEB, *n* = 86), Dai Chinese (CDX, *n* = 93), Utah residents with Northern and Western European ancestry (CEU, *n* = 99), Han Chinese (CHB, *n* = 103), Southern Han Chinese (CHS, *n* = 105), Colombian (CLM, *n* = 94), Esan (ESN, *n* = 99), Finnish (FIN, *n* = 99), British (GBR, *n* = 91), Gujarati (GIH, *n* = 103), Gambian Mandinka (GWD, *n* = 113), Iberian (IBS, *n* = 107), Telugu (ITU, *n* = 102), Japanese (JPT, *n* = 104), Kinh Vietnamese (KHV, *n* = 99), Luhya (LWK, *n* = 99), Mende (MSL, *n* = 85), Mexican Ancestry (MXL, *n* = 64), Peruvian (PEL, *n* = 85), Punjabi (PJL, *n* = 96), Puerto Rican (PUR, *n* = 104), Tamil (STU, *n* = 102), and Toscani (TSI, *n* = 107) [1]. **A** Admixture plot at K = 6. **B** Admixture plot at K = 10. **C** Admixture plot at K = 16; the lower panel displays a zoomed-in view of selected population segments. Individuals are grouped by population labels and sorted within each group according to the dominant ancestry proportion

Admixture barplots provide an intuitive summary of inferred ancestry components across individuals, enabling exploration of shared genetic backgrounds and population structure. Patterns of mixed ancestry across groups can suggest historical gene flow or recent admixture, while homogeneous profiles may reflect relative genetic continuity. Visualizing these proportions is particularly useful for communicating complex population histories and for comparing results across different values of K. The *admix* module visualizes admixture proportions across individuals, utilizing customized colored bar charts that facilitate a visual assessment of ancestry composition among populations. In this module, the input format is defined as a dataframe that includes population labels, individual IDs, and ancestry proportions. To show individual IDs, “--sl” option is specified (Fig. 6). Additionally, an ascending sorting approach was applied to sort individuals according to their ancestry proportional assignment (i.e., their most likely ancestry group).

### Manhattan plot module (*manplot*)

**Fig. 7:**
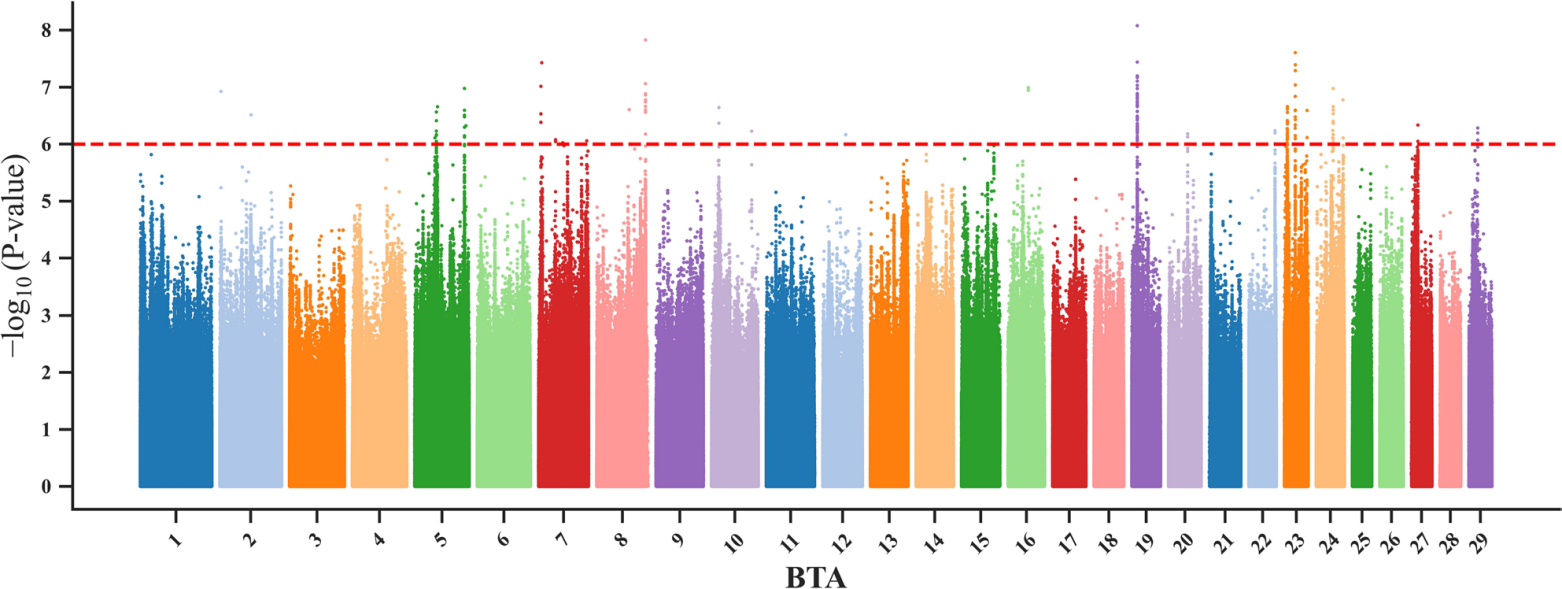
Manhattan plot generated using the manplot module of the GENOVIS package, based on publicly available GWAS results for heifer pregnancy success of 1,218 Australian Brahmans cattle (hybrid *Bos taurus/indicus*) and including 32,198,626 SNPs [10]. The red line is a suggestive line used to highlight SNPs with -log_10_(*P*-value) > 6

The *manplot* offers a flexible and automated framework to visualize Manhattan plots across various genetic analyses, such as GWAS, selection signatures, and nucleotide diversity. This module takes a tabular input (“--d”) containing chromosome names, base-pair position, and associated statistical values, and then automatically calculates cumulative genomic coordinates to arrange chromosomes sequentially along the x-axis. Users can specify two suggestive threshold lines (“--sug1” and “--sug2”) to highlight SNPs that exceed certain threshold levels (Fig. 7). Customization options include color palettes from matplotlib (“--c”), the number of colors (“--nc”), alpha blending (“--a”; a float value between 0 and 1), and point size (“--s”). For more details on colormaps in matplotlib, see here (https://matplotlib.org/stable/users/explain/colors/colormaps.html).

Compared to existing tools such as qqman [5], CMplot [43], and custom-made scripts using ggplot2 [64], which often require coding and offer limited flexibility, *manplot* offers a fully automated and customizable framework designed explicitly for genome-wide selection and association analyses (Fig. 7). This visualization facilitates the detection of localized signals against the genomic background by summarizing association or selection statistics across the genome. These patterns can reflect adaptive processes or trait-linked regions to guide further biological investigation.

To benchmark GENOVIS against other existing tools, we compared its functionality and workflow requirements with commonly used visualization tools in population genomics. Most existing custom scripts based on visualization libraries such as ggplot2 [64] and matplotlib [44] require preprocessing (e.g., binning for SNP density, calculating cumulative genomic coordinates for Manhattan plots, and annotating population labels), which relies on custom coding and reduces reproducibility. In contrast, GENOVIS automates all essential preprocessing steps internally. In comparison, some R packages, such as qqman [5] and CMplot [43], support only one or two plot types, whereas GENOVIS supports six common types of population genomics visualizations within a single framework. This integration simplifies the workflow and improves reproducibility. Additionally, GENOVIS incorporates systematic input validation, such as checking for file structure, dimensional consistency, missing values, and sample-label matching, which helps prevent errors in the visualization workflow. Such validation may not be considered in user-written custom scripts based on libraries like matplotlib [44] or ggplot2 [64], where input handling typically depends on manual coding decisions. A current limitation is that GENOVIS focuses exclusively on downstream visualization and does not implement upstream analyses, such as PCA, ROH, or admixture analyses, which external tools must perform.

Each plotting routine in GENOVIS is accessible via “argparse”, ensuring both scriptability and ease of use. Additionally, for users preferring a graphical environment, GENOVIS has a GUI version that operates without the need for coding (by running “genovis-gui” in Linux/Mac terminal or the Windows Command Prompt/PowerShell). Output files can be rendered in a wide range of high-quality bitmap and/or vector graphic formats (pdf, svg, svgz, png, tif, tiff, jpg, jpeg, eps, pgf, ps, raw, rgba, webp), making GENOVIS particularly ideal to generate publication-quality figures for the aforementioned population genomics analyses. From an architectural perspective, GENOVIS is structured as a hierarchical set of entry points within a parent script (genovis or genovis-gui), which offers a central CLI. This parent script sends user requests to the related plotting module. This approach enhances reproducibility, facilitates integration across analyses, and remains user-friendly, even for users with minimal programming experience. In addition to this architectural organization, GENOVIS can be integrated with standard population genomics pipelines (for PCA, ROH or CNV detection, relationship construction, admixture, GWAS) due to its compatibility with commonly used output formats produced by tools such as PLINK [22]. However, some modules require minor preprocessing steps (such as column selection). These steps are minimal, simple, and reproducible, making GENOVIS well-suited for incorporation into existing pipelines.

In summary, GENOVIS offers a unified, comprehensive Python framework for generating six key population genomics visualizations, including SNP density (*mapden*), runs of homozygosity (*rohpainter*), relationship heatmaps (*relmap*), 3D PCA (*pca3d*), admixture barplots (*admix*), and Manhattan plots (*manplot*). By supporting standard input formats and offering extensive customization through both a unified CLI and GUI interfaces, this tool eliminates the need for using multiple fragmented tools and complex scripting. Its ability to generate publication-ready bitmap or vector graphics, with customizable fonts (type, size) and color maps, makes it a powerful solution for synchronizing and refining visual outputs. Ultimately, GENOVIS enhances reproducibility, clarity, and workflow efficiency in population genomic data visualization, while remaining accessible to users with limited programming experience.

## Supplementary Information

Supplementary Material 1.

## Data Availability

Source of GENOVIS is downloadable from [https://pypi.org/project/genovis] or [https://github.com/Siavash-cloud/GENOVIS]. The user guide of GENOVIS is available at [https://siavash-cloud.github.io/GENOVIS] and [https://github.com/Siavash-cloud/GENOVIS].

## Abbreviations

2D and 3D PCA: Two- and Three-dimensional principal component analysis
ACB: African Caribbean
ASW: African Ancestry Southwest
BEB: Bengali
BSW: Brown Swiss
CDX: Dai Chinese
CEU: Utah residents (CEPH) with Northern and Western European ancestry
CHB: Han Chinese
CHS: Southern Han Chinese
CLI: Command line interface
CLM: Colombian
DEC: Dengchuan
ESN: Esan
FIN: Finnish
GBR: British
GIH: Gujarati
GUI: Graphical user interface
GWAS: Genome-wide association study
GWD: Gambian Mandinka
IBS: Iberian
ITU: Telugu
JPT: Japanese
KHV: Kinh Vietnamese
LWK: Luhya
MSL: Mende
MXL: Mexican Ancestry
NEL: Nelore
PCA: Principal component analysis
PyPI: Python Package Index
PEL: Peruvian
PJL: Punjabi
PUR: Puerto Rican
RGU: Red Angus
ROH: Runs of homozygosity
SHK: Sheko
SNP: Single-nucleotide polymorphism
STU: Tamil
TSI: Toscani
YAK: Yakutian cattle
YRI: Yoruba
ZAR: Arabic zebu
