# Supplementary material for: GENOVIS: a Python package for the visualization of population genetic analyses
Source: BMC Genomics. 2026 Feb 10;27:190. doi: 10.1186/s12864-026-12598-x (PMC12903657; doi:10.1186/s12864-026-12598-x)
Supplement: Supplementary file 1 — Supplementary Material 1. [file 12864_2026_12598_MOESM1_ESM.docx]

**Supplementary information 1**

**1. Dataset for *mapden* module**

We downloaded the publicly available map file of GeneSeek Equine SNP 65 Bead Chip (65,157 SNPs, assembly: EquCab 2.0) panel from [https://webserver.ibba.cnr.it/SNPchimp](https://webserver.ibba.cnr.it/SNPchimp/) to test *mapden* module. Consequently, heatmaps of SNP densities were generated using the following commands in GENOVIS:

| # for Equine panel  genovis mapden --m GeneSeek_EquineSNP65_BeadChip_65157_EquCab2.0.map --i index_EquCab2.0.txt --o GeneSeek_EquineSNP65_BeadChip_65157_EquCab2.0 --b 1 --Chr ECA --ft svg --f "Times New Roman" --c Reds --mode solid --Chrfs 8 |
| --- |

**2. Dataset for *relmap* module**

To construct a relationship matrix, we downloaded a publicly available genotypes (<https://doi.org/10.5061/dryad.37pvmcvqr>) from four Persian horse populations (Asil, Caspian, Kurdish, and Turkoman). This dataset has passed quality control steps as defined by Mousavi et al. (2023) [1]. Then, the relationship matrix construction was done by PLINK software (version 2.0.0) [2] as defined below:

| ./plink2 --bfile Data_Final --chr-set 31 --make-rel square --out GRM_horse |
| --- |

After removing the header of the index file generated (“*.id”) by PLINK software (version 2.0.0) [2], a heatmap plot based on this matrix was generated using *relmap* module in GENOVIS as defined below:

| genovis relmap --relfile GRM_horse.rel --matindex GRM_horse.rel.id --mode solid --x 8 --y 8 --pfs 14 --sl true --c plasma_r --xyfs 3 --o Persian_horse_relmap --rf mat --ft svg --f "Times New Roman" --av true |
| --- |

Moreover, a heatmap plot based on the averages of relationships between/within populations was generated (with averages calculated by applying “--av true” in the aforementioned command) as defined below:

| genovis relmap --relfile Persian_horse_relmap_pop_avg.col --mode solid --x 10 --y 8 --pfs 14 --c Reds --o Persian_horse_relmap_AVERAGES --rf col --ft svg --f "Times New Roman" --xyfs 15 --a true --afs 14 |
| --- |

**3. Dataset for *pca3d* module**

For visualizing PCA plots, we downloaded PCA results achieved by Chen et al. (2022) [3] (<https://doi.org/10.6084/m9.figshare.20294343.v1>) for two goat populations (Nubian and Yunling). Then, the dataset was used directly to generate 3D-PCA and 2D-PCA plots as defined below:

| #3D-PCA(--azim 65 --elev 25)  genovis pca3d --evec pop.pca.eigenvec --eval pop.pca.eigenval --dim 3d --s 70 --mode solid --fs 12 --fp 1 --sp 2 --tp 3 --ft svg --o 3dPCA_65_25 --f "Times New Roman" --x 8 --y 5 --azim 65 --elev 25  #3D-PCA(--azim 45 --elev 45)  genovis pca3d --evec pop.pca.eigenvec --eval pop.pca.eigenval --dim 3d --s 70 --mode solid --fs 12 --fp 1 --sp 2 --tp 3 --ft svg --o 3dPCA_45_45 --f "Times New Roman" --x 8 --y 5 --azim 45 --elev 45  #3D-PCA(--azim 25 --elev 65)  genovis pca3d --evec pop.pca.eigenvec --eval pop.pca.eigenval --dim 3d --s 70 --mode solid --fs 12 --fp 1 --sp 2 --tp 3 --ft svg --o 3dPCA_25_65 --f "Times New Roman" --x 8 --y 5 --azim 25 --elev 65  #2D-PCA (PC1 and PC2)  genovis pca3d --evec pop.pca.eigenvec --eval pop.pca.eigenval --dim 2d --s 70 --mode solid --fs 12 --fp 1 --sp 2 --ft svg --o 2dPCA_PC1_PC2 --f "Times New Roman" --x 8 --y 5  #2D-PCA (PC1 and PC3)  genovis pca3d --evec pop.pca.eigenvec --eval pop.pca.eigenval --dim 2d --s 70 --mode solid --fs 12 --fp 1 --sp 3 --ft svg --o 2dPCA_PC1_PC3 --f "Times New Roman" --x 8 --y 5  #2D-PCA (PC2 and PC3)  genovis pca3d --evec pop.pca.eigenvec --eval pop.pca.eigenval --dim 2d --s 70 --mode solid --fs 12 --fp 2 --sp 3 --ft svg --o 2dPCA_PC2_PC3 --f "Times New Roman" --x 8 --y 5 |
| --- |

**4. Dataset for *admix* module**

We downloaded the admixture results of Auton et al. (2015) study [4] ([https://ftp.1000genomes.ebi.ac.uk/vol1/ftp/release/20130502/supporting/admixture_files](https://ftp.1000genomes.ebi.ac.uk/vol1/ftp/release/20130502/supporting/admixture_files/)) to generate the admixture plot for 26 human populations (Table 1) at K={6, 10, 16} by applying the *admix* module in GENOVIS. The codes for generating admixture plots were:

| #admix (at K=6)  genovis admix --d K6_1000genome.Q --c tab20 --fs 40 --lws 0.7 --x 70 --y 5 --o admix_K6 --ft jpg --f "Times New Roman" --dpi 800 --sl false --mode solid  #admix (at K=10)  genovis admix --d K10_1000genome.Q --c tab20 --fs 40 --lws 0.7 --x 70 --y 5 --o admix_K10 --ft jpg --f "Times New Roman" --dpi 800 --sl false --mode solid  #admix (at K=16)  genovis admix --d K16_1000genome.Q --c tab20 --fs 40 --lws 0.7 --x 70 --y 5 --o admix_K16 --ft jpg --f "Times New Roman" --dpi 800 --sl true --mode solid --xt 2 |
| --- |

**Table 1.** List of admixture results (at K={6, 10, 16}) of 26 human populations (n=2,504) downloaded from <https://ftp.1000genomes.ebi.ac.uk/vol1/ftp/release/20130502/supporting/admixture_files>.

| Population | Population flag | # Genotyped individuals |
| --- | --- | --- |
| African Caribbean | ACB | 96 |
| African Ancestry Southwest | ASW | 61 |
| Bengali | BEB | 86 |
| Dai Chinese | CDX | 93 |
| Utah residents (CEPH) with Northern and Western European ancestry | CEU | 99 |
| Han Chinese | CHB | 103 |
| Southern Han Chinese | CHS | 105 |
| Colombian | CLM | 94 |
| Esan | ESN | 99 |
| Finnish | FIN | 99 |
| British | GBR | 91 |
| Gujarati | GIH | 103 |
| Gambian Mandinka | GWD | 113 |
| Iberian | IBS | 107 |
| Telugu | ITU | 102 |
| Japanese | JPT | 104 |
| Kinh Vietnamese | KHV | 99 |
| Luhya | LWK | 99 |
| Mende | MSL | 85 |
| Mexican Ancestry | MXL | 64 |
| Peruvian | PEL | 85 |
| Punjabi | PJL | 96 |
| Puerto Rican | PUR | 104 |
| Tamil | STU | 102 |
| Toscani | TSI | 107 |
| Yoruba | YRI | 108 |

**5. Dataset for** ***rohpainter* module**

We downloaded the genotypes of cattle populations (Table 2) published by previous studies [5-8] from <http://widde.toulouse.inra.fr>.

**Table 2.** List of cattle breeds (n=7) and number of individuals (n=184) genotyped for 52,515 autosomic SNPs using Illumina Bovine SNP 50 v1.

| Population | Population flag | # Genotyped individuals |
| --- | --- | --- |
| Brown Swiss | BSW | 24 |
| Dengchuan | DEC | 31 |
| Nelore | NEL | 24 |
| Red Angus | RGU | 15 |
| Sheko | SHK | 20 |
| Yakutian cattle | YAK | 40 |
| Arabic Zebu | ZAR | 35 |

Here, we filtered SNPs with minor allele frequency < 0.01, SNP calling rate < 0.90, and Hardy-Weinberg equilibrium P-value < 10^-6^. Additionally, samples with less than 90% genotype call rate were discarded from downstream analyses using PLINK software (version 1.9) [2]:

| ./plink --file cattle__52515variants__189individuals --chr 1-29 --maf 0.01 --make-bed --cow --out maf  ./plink --bfile maf --cow --geno 0.1 --make-bed --out maf_geno  ./plink --bfile maf_geno --make-bed --cow --hwe 0.000001 --out maf_geno_hwe  ./plink --bfile maf_geno_hwe --recode ped --cow --mind 0.1 --out maf_geno_hwe_mind |
| --- |

The detectRUNS package [9] was used in the R environment [10] to detect runs of homozygote regions as:

| ROH<-slidingRUNS.run("maf_geno_hwe_mind.ped"  ,"maf_geno_hwe_mind.map",  minSNP = 15,  minLengthBps=500000,  maxGap=10^6,  minDensity = 1/100,  maxMissWindow=1,  maxOppWindow=1)  ROH<-ROH[,c(1,2,3,5,6)]  write.table(ROH,"all_rohpainter.txt",col.names=FALSE,row.names=FALSE,quote=FALSE) |
| --- |

Then, runs of homozygote regions were plotted using the *rohpainter* module in GENOVIS (command-line based) as:

| genovis rohpainter --d all_rohpainter.txt --i index_UMD3.1.txt --t 0.7 --yt 3 --tc black --o ROH_cattle --y 10 --x 10 --f "Times New Roman" --tw 0.5 --c Set1 --mode solid --ft svg |
| --- |

**6. Dataset for *manplot* module**

For generating a Manhattan plot, GWAS results of a former study on heifer pregnancy success by Forutan et al. (2024) [11] were downloaded from <https://doi.org/10.6084/m9.figshare.25807276>. After transforming P-values (for 32,198,626 SNPs) to -log_10_(P-value) in the R environment (version 4.4.1) [10], *manplot* module was used to plot a Manhattan plot, following the command in the Linux terminal:

| genovis manplot --d GWAS_preg_st_FOROUTAN_32198626SNPs.txt --mode solid --c tab20 --nc 10 --a 1 --x 10 --y 4 --s 0.25 --sug1 6 --sug1c red --f "Times New Roman" --xlab BTA --ylab "$-\log_{10}(\mathrm{P\text{-}value})$" --o Manhattan_plot_cattle --dpi 800 --ft jpg |
| --- |

1. Mousavi SF, Razmkabir M, Rostamzadeh J, Seyedabadi H-R, Naboulsi R, Petersen JL, Lindgren G: **Genetic diversity and signatures of selection in four indigenous horse breeds of Iran**. *Heredity* 2023, **131**(2):96-108.

2. Purcell S, Neale B, Todd-Brown K, Thomas L, Ferreira MA, Bender D, Maller J, Sklar P, de Bakker PI, Daly MJ *et al*: **PLINK: a tool set for whole-genome association and population-based linkage analyses**. *American journal of human genetics* 2007, **81**(3):559-575.

3. Chen Y, Li R, Sun J, Li C, Xiao H, Chen S: **Genome-Wide Population Structure and Selection Signatures of Yunling Goat Based on RAD-seq**. In: *Animals.* vol. 12; 2022: 2401.

4. Auton A, Abecasis GR, Altshuler DM, Durbin RM, Abecasis GR, Bentley DR, Chakravarti A, Clark AG, Donnelly P, Eichler EE *et al*: **A global reference for human genetic variation**. *Nature* 2015, **526**(7571):68-74.

5. Matukumalli LK, Lawley CT, Schnabel RD, Taylor JF, Allan MF, Heaton MP, O'Connell J, Moore SS, Smith TPL, Sonstegard TS *et al*: **Development and Characterization of a High Density SNP Genotyping Assay for Cattle**. *PLOS ONE* 2009, **4**(4):e5350.

6. Iso-Touru T, Tapio M, Vilkki J, Kiseleva T, Ammosov I, Ivanova Z, Popov R, Ozerov M, Kantanen J: **Genetic diversity and genomic signatures of selection among cattle breeds from Siberia, eastern and northern Europe**. *Animal Genetics* 2016, **47**(6):647-657.

7. Gao Y, Gautier M, Ding X, Zhang H, Wang Y, Wang X, Faruque MDO, Li J, Ye S, Gou X *et al*: **Species composition and environmental adaptation of indigenous Chinese cattle**. *Scientific Reports* 2017, **7**(1):16196.

8. Gautier M, Flori L, Riebler A, Jaffrézic F, Laloé D, Gut I, Moazami-Goudarzi K, Foulley J-L: **A whole genome Bayesian scan for adaptive genetic divergence in West African cattle**. *BMC Genomics* 2009, **10**(1):550.

9. Biscarini F, Cozzi P, Gaspa G, Marras G: **Detect runs of homozygosity and runs of heterozygosity in diploid genomes**. 2019.

10. Team RC: **R: A Language and Environment for Statistical Computing**. 2024.

11. Forutan M, Engle BN, Chamberlain AJ, Ross EM, Nguyen LT, D’Occhio MJ, Snr AC, Kho EA, Fordyce G, Speight S *et al*: **Genome-wide association and expression quantitative trait loci in cattle reveals common genes regulating mammalian fertility**. *Communications Biology* 2024, **7**(1):724.
